# THE EFFECT OF ETHNIC ENCLAVE ON COGNITION AND EVERYDAY FUNCTIONING DIFFERS ACROSS NEIGHBORHOOD DISADVANTAGE

**DOI:** 10.1093/geroni/igae098.0484

**Published:** 2024-12-31

**Authors:** Alexandra Clark, Emily Morriss, Jeremy Grant, Darlingtina Esiaka, Kristine Ajrouch, Wassim Tarraf

**Affiliations:** The University of Texas Austin, Austin, Texas, United States; University of Michigan, Ann Arbor, Michigan, United States; University of Florida, Gainseville, Florida, United States; University of Kentucky College of Medicine, University of Kentucky, Kentucky, United States; University of Michigan, Ann Arbor, Michigan, United States; Wayne State University, Detroit, Michigan, United States

## Abstract

Ethnic enclaves represent concentrated geographic areas where Hispanic community members reside in close proximity to same group peers. Previous research has revealed evidence of a “barrio advantage” where living in an ethnic enclave is associated with better health outcomes. However, there is some evidence to suggest (1) the protective effects of enclave may be moderated by neighborhood-level socioeconomic factors and (2) the impact of ethnic enclave on cognitive and functional outcomes remain poorly understood. The present study leveraged data from the Hispanic Established Populations for the Epidemiological Study of the Elderly to examine the effects of ethnic enclaves on cognitive and functional outcomes. We examined associations between tract-level segregation (Hispanic-Specific Getis-Ord Gi*) and baseline cognitive and functional outcomes, as well as change over time. Analyses included cluster adjusted linear regression models sequentially adjusted for neighborhood and individual level sociodemographic measures. Findings showed a positive association between enclave residence and baseline cognitive status, controlling for neighborhood and individual level socioeconomic disadvantage. The enclave effects did not extend to baseline functional status. Despite positive enclave effects at baseline, enclave residence was associated with faster rates of cognitive and functional decline. Markers of socioeconomic disadvantage were not consistent modifiers of associations between the enclave effect and cognitive and functional outcomes. Results will be discussed within the ecological system theory to emphasize environmental contexts are important for late life cognitive and functional outcomes, and further highlight potential points of intervention that may promote equitable aging outcomes in Hispanic older adults.

